# Defeating meningitis by 2030 – an ambitious target

**DOI:** 10.1093/trstmh/trab133

**Published:** 2021-09-02

**Authors:** Brian Greenwood, Samba Sow, Marie-Pierre Preziosi

**Affiliations:** Faculty of Infectious and Tropical Diseases, London School of Hygiene and Tropical Medicine, Keppel St., London WC1E 7HT, UK; Centre for Vaccine Development, Avenue Mohamed VI, Djikoni, 251, Bamako, Mali; Department of Immunization, Vaccines and Biologicals, World Health Organization, 20 Avenue Appia, 1211 Geneva 27, Switzerland

**Keywords:** acute bacterial meningitis, defeating meningitis by 2030, roadmap

## Abstract

Acute bacterial meningitis remains a major cause of mortality and morbidity, especially in lower-income countries. Thus, in 2017, a group of people concerned with this continuing problem came together to plan a way forward. A task force was established, a baseline situation analysis undertaken and a road map for a new initiative ‘Defeating Meningitis by 2030’ prepared. This road map will be launched officially in September 2021. Additional finances for meningitis control will be needed, together with the support of many different institutions and people with different skills, if the ‘Defeating Meningitis by 2030’ initiative is to achieve its ambitious goals.

Substantial progress has been made in the prevention of acute bacterial meningitis (ABM) during the past few decades, but this group of infections still remains a major cause of mortality and morbidity, especially in low- and middle-income countries (LMICs). Determining the exact burden of ABM is challenging and different methods of evaluation provide varying information, especially the number of cases attributable to individual pathogens.^1^ However, a credible analysis suggested that there were approximately 290 000 deaths from meningitis in 2017.^2^ This number increases substantially if cases, and deaths, from ABM and neonatal meningitis are combined. Many people affected by ABM, especially that caused by *Streptococcus pneumoniae*, are left with severe neurological sequelae^3^ and in many LMICs these patients have very limited ongoing support. Despite these continuing daunting numbers, there is strong evidence that the incidence of ABM has fallen during the past few decades.^1^ This has been achieved primarily through vaccination with polysaccharide/protein conjugate vaccines, which are immunogenic in young children and prevent nasopharyngeal carriage, thus interrupting transmission of infection. Success was achieved first in protecting against *Haemophilus influenzae* type b (Hib) infections and then against infections by some *S. pneumoniae* serotypes and some serogroups of *Neisseria meningitidis*. More recently, the development of a protein vaccine against *N. meningitidis* group B has been a further success.^4^ Progress has also been made in other areas, such as increasing population awareness of the clinical features of meningitis and the need to seek treatment rapidly, and in the development of polymerase chain reaction methods of diagnosis that can be implemented at scale. However, despite these advances, mortality and morbidity from many types of ABM remain unacceptability high and many people affected by ABM, especially that caused by *S. pneumoniae*, are left with serious long-term sequelae and have little or no access to appropriate support in many LMICs.

The success of vaccination programmes has led to a perception in some organisations that the problem of ABM is over, but the numbers listed above show that this is not the case and that there is still much more that needs to be done to bring ABM under full control. There is no obvious single target that will lead to a marked decrease in the burden of ABM, with the possible exception of the development of an effective vaccine against group B *Streptococcus*,^5^ and progress will be needed on several different fronts at the same time if success in reducing the persisting burden of ABM is to be achieved. Recognising this challenge, a group of representatives of civil society and governments, together with scientists from a wide range of disciplines but all with an interest in preventing or improving the management of cases of meningitis, came together at a meeting held in Wilton Park, UK in May 2017 to discuss what could be done to achieve enhanced support for the control of ABM. This meeting led to the formation of a task force, led by the World Health Organization (WHO), but which includes individuals from many different countries and institutions and members with many different areas of expertise, to take on the challenge of leading and coordinating the activities needed to reduce the current burden of ABM.

The first activity of the task force was to undertake a baseline situation analysis^6^ that identified areas where further progress needs to be made to reduce the burden of ABM. This informed the development of a detailed road map ‘Defeating Meningitis by 2030’^7^ that sets out the broad range of activities that need to be undertaken, including priorities, targets and timelines that must be achieved if defeating meningitis by 2030 is to be achieved. The visionary goals of the ‘Defeating Meningitis by 2030’ road map are to, by 2030, eliminate bacterial meningitis epidemics, reduce cases of vaccine-preventable bacterial meningitis by 50% and deaths by 70% and reduce disability and improve quality of life after meningitis due to any cause.

Early versions of the roadmap were circulated widely for comments and discussed at global and regional meetings and with WHO member states before a final version of the roadmap was approved by the 73rd World Health Assembly in November 2020, when member states endorsed the first-ever resolution on meningitis prevention and control,^8^ with a request to the Director General to support its implementation. In September 2021, the roadmap will be formally launched by the WHO at a series of events. The roadmap is set out under five interconnected headings (pillars), each including several activities with their own strategic goals, key activities and milestones (Figure 1).

**Figure 1.**
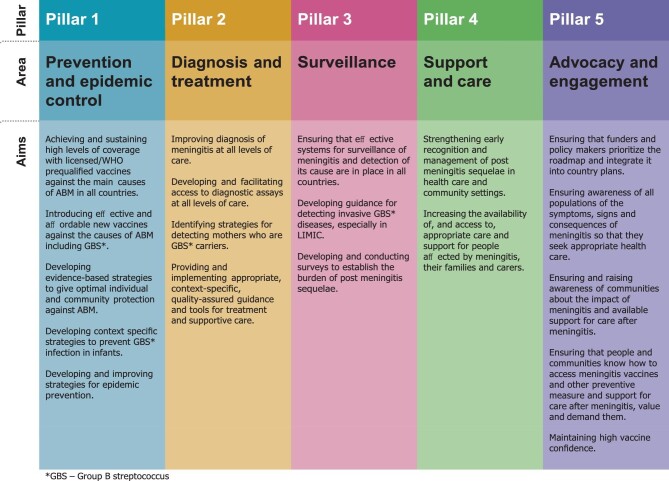
Pillars of the ‘Defeating Meningitis Roadmap by 2030’ Roadmap.

Different institutions have been given overall responsibility for moving forward individual components of the road map and the task force will continue to provide overall technical leadership and coordination. However, none of this will be possible unless enough resources are made available for the key activities proposed. Consequently, the WHO has established a Strategy Support Group of global health leaders who will assist in obtaining the political and financial support needed to take this initiative forward to a successful conclusion. A business plan to support the initiative is also being developed.

The coronavirus disease 2019 pandemic has had a complex effect on many infectious diseases, including ABM. Despite a decrease in vaccination coverage rates, there has been a reduction in the spread of many pathogens, including those responsible for pneumonia and ABM, due to lockdowns and other control measures.^9,10^ This short-term positive situation could lead to a dangerous disinterest in the fight against the pathogens responsible for ABM, which would compromise achievement of the objectives of the road map unless vigorous efforts are made to counter this complacency.

Acute bacterial meningitis is a global problem and one that is especially important in LMICs where many members of the Royal Society of Tropical Medicine and Hygiene (RSTMH) are based. Thus, through its membership, the RSTMH has an opportunity to contribute to the ‘Defeating Meningitis by 2030’ initiative and make it a success.
